# Supplementary material for: Parity-conserving Cooper-pair transport and ideal superconducting diode in planar germanium
Source: Nat Commun. 2024 Jan 2;15:169. doi: 10.1038/s41467-023-44114-0 (PMC10762135; doi:10.1038/s41467-023-44114-0)
Supplement: Supplementary file 1 — Supplementary Information [file 41467_2023_44114_MOESM1_ESM.pdf]

Supplementary Information for 'Parity-conserving Cooper-pair transport and ideal superconducting diode in planar Germanium'

Marco Valentini[1,*], Oliver Sagi[1], Levon Baghumyan[1], Thijs de Gijsel[1,2], Jason Jung[2], Stefano Calcaterra[3], Andrea Ballabio[3], Juan Aguilera Servin[1], Kushagra Aggarwal[1,4], Marian Janik[1], Thomas Adletzberger[1], Rubén Seoane Souto[5,6], Martin Leijnse[7], Jeroen Danon[8], Constantin Schrade[5], Erik Bakkers[2], Daniel Chrastina[3], Giovanni Isella[3], Georgios Katsaros[1,*]

[1]Institute of Science and Technology Austria, Am Campus 1, 3400 Klosterneuburg, Austria.
[2] Department of Applied Physics, Eindhoven University of Technology, Eindhoven, The Netherlands.
[3] L-NESS, Physics Department, Politecnico di Milano, via Anzani 42, 22100, Como, Italy.
[4] Department of Materials, University of Oxford, Oxford OX1 3PH, United Kingdom.
[5] Center for Quantum Devices, Niels Bohr Institute, University of Copenhagen, 2100 Copenhagen, Denmark.
[6] Instituto de Ciencia de Materiales de Madrid, Consejo Superior de Investigaciones Científicas (ICMM-CSIC), Madrid, Spain.
[7] NanoLund and Solid State Physics, Lund University, Box 118, 22100 Lund, Sweden.
[8] Department of Physics, Norwegian University of Science and Technology, NO-7491 Trondheim, Norway.
[*] Corresponding authors: marco.valentini@ist.ac.at and georgios.katsaros@ist.ac.at .

November 28, 2023

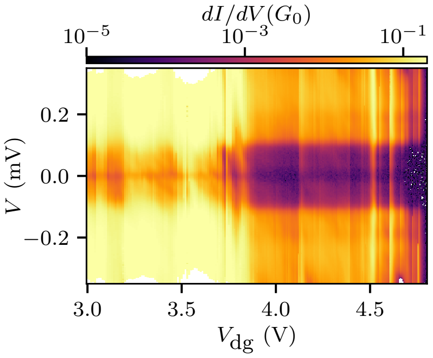

Figure S1: **Additional data for Fig.2.** dI/dV as a function of V and $V_{\rm dg}$ plotted in logarithmic scale for sample D5. The data for less positive $V_{\rm dg}$ show indications of quantum dots formation.

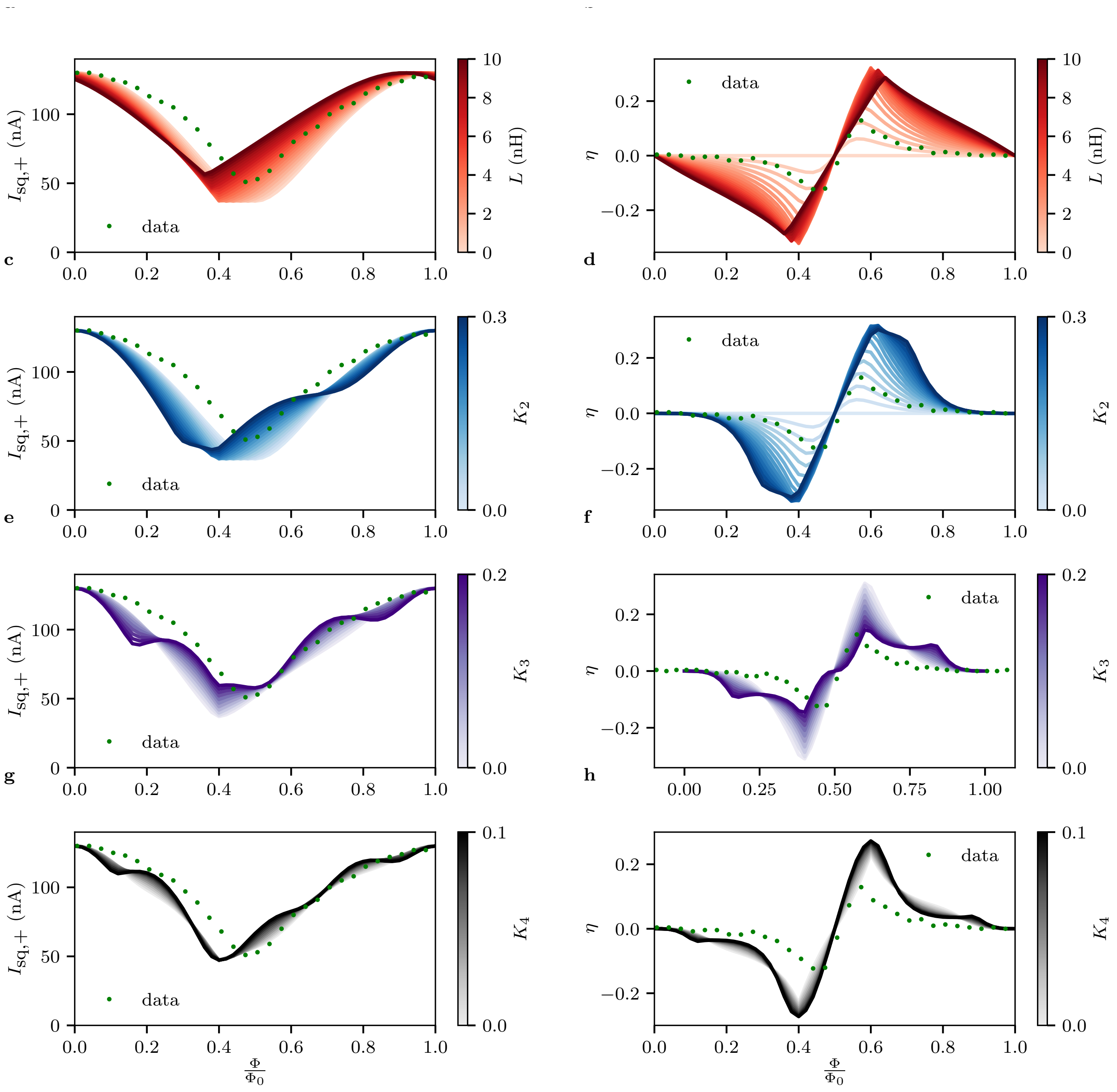

Figure S2: **SQUID behaviour for different cases.** Left column [right column] $I_{\mathrm{sq},+}$ [η] as a function of Φ. The green dots represent the experimental values extracted from Fig. 4**b**. **a** [**b**] represent the theoretical calculation for different values of L and ignoring higher harmonic contribution. The blue traces in **c-d** represent the theoretical calculation using $L = 0$, $K_1 = 1 - K_2, K_3 = K_4 = 0$ and K_2 varies from 0 to 0.3. The purple traces in **e-f** are for $L = 0$, $K_1 = 0.83 - K_3, K_2 = 0.17, K_4 = 0$ and K_3 varies from 0 to 0.2. The black traces in **g-h** are for $L = 0$, $K_1 = 0.73 - K_4, K_2 = 0.17, K_3 = 0.1$ and K_4 varies from 0 to 0.1.

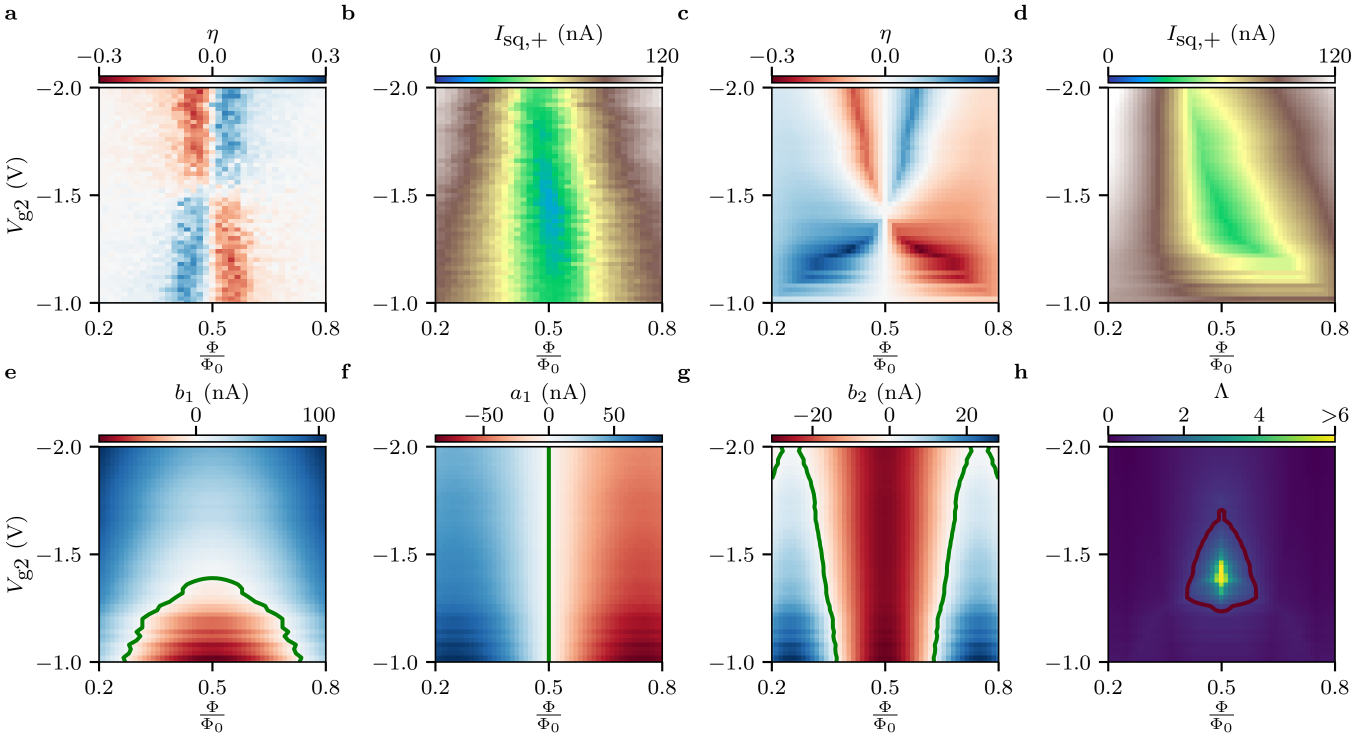

Figure S3: **Expected results with asymmetric harmonic contributions.** **a** [**b**] shows the same plot as Fig. 4**e** [Fig. 4**f**]. **c** [**d**] Theoretical prediction of **a** [**b**], where, like in the main text, it was assumed that $L = 110\,\mathrm{pH}$ and $V_{g1} = -1.5\,\mathrm{V}$. The harmonic contributions of I_{JJ2} are assumed to be $K_1 = 0.7, K_2 = 0.1, K_3 = 0.1$ and I_{JJ1} was assumed to have more higher harmonic contribution, namely I_{JJ1} has $K_1 = 0.6, K_2 = 0.2, K_3 = 0.1$. **e-f** First-order harmonics contribution as a function of V_{g2} and Φ. Both terms vanish close to the balanced point and at $\frac{\Phi}{\Phi_0} = 0.5$. **g** Sinusoidal second harmonic contribution as a function of V_{g2} and Φ. Importantly, b_2 never vanishes at $\frac{\Phi}{\Phi_0} = 0.5$. **h** Ratio between second and first harmonic as a function of V_{g2} and Φ. For this situation, the second component would dominate the CPR not only at the sweet spot. Therefore a small asymmetry between the harmonic contributions would not change the fact that the second harmonic contribution never vanishes at $\frac{\Phi}{\Phi_0} = 0.5$. In **c-h** the same parameters have been used.

sample	f_r(GHz)	Q_e	$Q_{i,\langle n_{ph}\rangle=0.1}$	$Q_{i,\langle n_{ph}\rangle=1}$	$Q_{i,\langle n_{ph}\rangle=10}$	$Q_{i,\langle n_{ph}\rangle=100}$
without QW	4.01	6150 ± 150	2800 ± 200	6000 ± 300	13000 ± 600	30000 ± 3000
without QW	4.04	6300 ± 150	n.a.	6270 ± 130	10000 ± 130	15700 ± 170
without QW	4.18	3250 ± 70	n.a.	9240 ± 350	15350 ± 450	26350 ± 635
without QW	4.23	3380 ± 40	n.a.	5670 ± 140	8220 ± 125	11000 ± 175
without QW	4.27	3800 ± 150	2700 ± 200	7000 ± 300	15000 ± 800	26000 ± 3000
without QW	4.60	2750 ± 150	3800 ± 200	8800 ± 300	17000 ± 500	24000 ± 1000
without QW	4.66	4000 ± 300	5200 ± 500	10000 ± 500	21000 ± 700	40000 ± 2000
without QW	4.77	2000 ± 40	n.a.	7780 ± 200	11900 ± 400	17100 ± 1120
without QW	4.84	1370 ± 15	n.a.	7600 ± 360	12220 ± 1220	18350 ± 3000
without QW	4.91	2500 ± 200	5000 ± 400	10000 ± 600	17000 ± 1000	27000 ± 2000
without QW	5.02	2100 ± 70	3100 ± 100	4300 ± 100	6560 ± 240	9000 ± 725
without QW	5.24	1550 ± 20	4100 ± 175	5340 ± 160	7430 ± 300	9640 ± 600
without QW	5.72	1660 ± 25	4300 ± 135	5500 ± 145	7660 ± 260	10200 ± 930
without QW	5.78	1365 ± 20	4830 ± 180	5950 ± 140	8230 ± 220	10270 ± 500
without QW	6.00	1700 ± 20	5250 ± 220	6300 ± 150	9060 ± 230	12580 ± 500
D5	4.04	4740 ± 800	n.a.	5365 ± 225	8920 ± 550	14360 ± 1240
D5	4.13	3200 ± 215	n.a.	6400 ± 260	10530 ± 825	15400 ± 1000
D5	4.22	3300 ± 130	n.a.	3230 ± 230	11000 ± 610	15750 ± 1160
D5	4.35	3085 ± 215	n.a.	5850 ± 230	10150 ± 570	15200 ± 1200
D5	4.39	1440 ± 55	n.a.	5690 ± 220	10400 ± 680	14200 ± 1750
D5	4.43	2100 ± 100	n.a.	5500 ± 210	8900 ± 470	13040 ± 720
D5	4.52	1680 ± 240	n.a.	5230 ± 510	6880 ± 1060	10000 ± 3050
D5	4.56	2450 ± 170	n.a.	7100 ± 220	12600 ± 1530	18150 ± 3700
D5	4.61	1250 ± 50	n.a.	6500 ± 400	20000 ± 4000	36000 ± 17000
D5	4.73	800 ± 20	4000 ± 200	7600 ± 500	14000 ± 2000	23000 ± 5000
D5	5.23	770 ± 25	2730 ± 200	4545 ± 340	6980 ± 1230	8530 ± 1400
D5	5.45	740 ± 25	3600 ± 200	5500 ± 200	10000 ± 900	17000 ± 1000
D5	5.83	970 ± 20	1645 ± 70	2160 ± 70	2680 ± 100	2910 ± 170
D5	5.97	630 ± 10	n.a.	2900 ± 150	3400 ± 100	4000 ± 200
D60	4.47	3220 ± 50	n.a.	4460 ± 75	5660 ± 50	6600 ± 110
D60	4.70	2870 ± 50	n.a.	3365 ± 65	4130 ± 90	4830 ± 110
D60	4.98	2745 ± 145	2700 ± 75	3200 ± 35	3700 ± 30	4100 ± 65
D60	5.09	2610 ± 160	2910 ± 130	3175 ± 55	3570 ± 65	4000 ± 130
D60	5.24	2100 ± 30	2440 ± 60	2870 ± 45	3260 ± 50	3470 ± 60
D60	5.34	683 ± 5	2300 ± 80	2555 ± 35	2840 ± 50	3070 ± 60
D60	5.68	627 ± 3	2040 ± 70	2240 ± 30	2530 ± 20	2670 ± 20
D60	6.14	730 ± 10	1740 ± 60	1930 ± 40	2100 ± 40	2200 ± 50

Table S1: **Summary of the resonator data.** Resonance frequency f_r, external quality factor Q_e and internal quality factor Q_i for different average photon number $\langle n_{ph}\rangle$ extracted using an algebraic fit of the parameter S_{21} [1]. The values reported here are average values and the error is given by the standard deviation. The error of f_r is not reported because it is smaller than 1 MHz. n.a. stands for not available. We note that higher frequency resonators show lower Q_i.

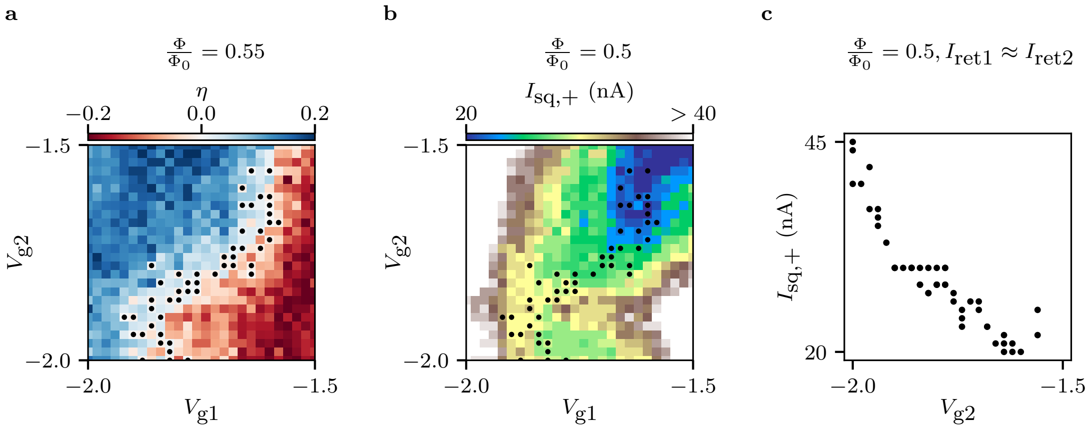

Figure S4: **Procedure to identify and quantify the region of the sweet spot.** **a** η for $\frac{\Phi}{\Phi_0} = 0.55$ as a function of V_{g1} and V_{g2}. If $|V_{g2}| > |V_{g1}|$ [$|V_{g2}| < |V_{g1}|$] $\eta < 0$ [$\eta > 0$]. Importantly, η vanishes along the diagonal line, i.e. if $V_{g1} \approx V_{g2}$ which corresponds to $I_{ret1} \approx I_{ret2}$. The black dots indicate the points at which $|\eta| < 0.015$. Once the $I_{ret1} \approx I_{ret2}$ region is identified, we move to $\frac{\Phi}{\Phi_0} = 0.5$ in order to be able to assess the sweet spot regime. **b** shows $I_{sq,+}$ as a function of V_{g1} and V_{g2}. $I_{sq,+}$ is finite even when the junctions are balanced, see black spots. **c** $I_{sq,+}$ as a function of V_{g2} in the sweet spot configuration. The identification of the balanced region cannot be carried out at half flux quantum because η is always zero.

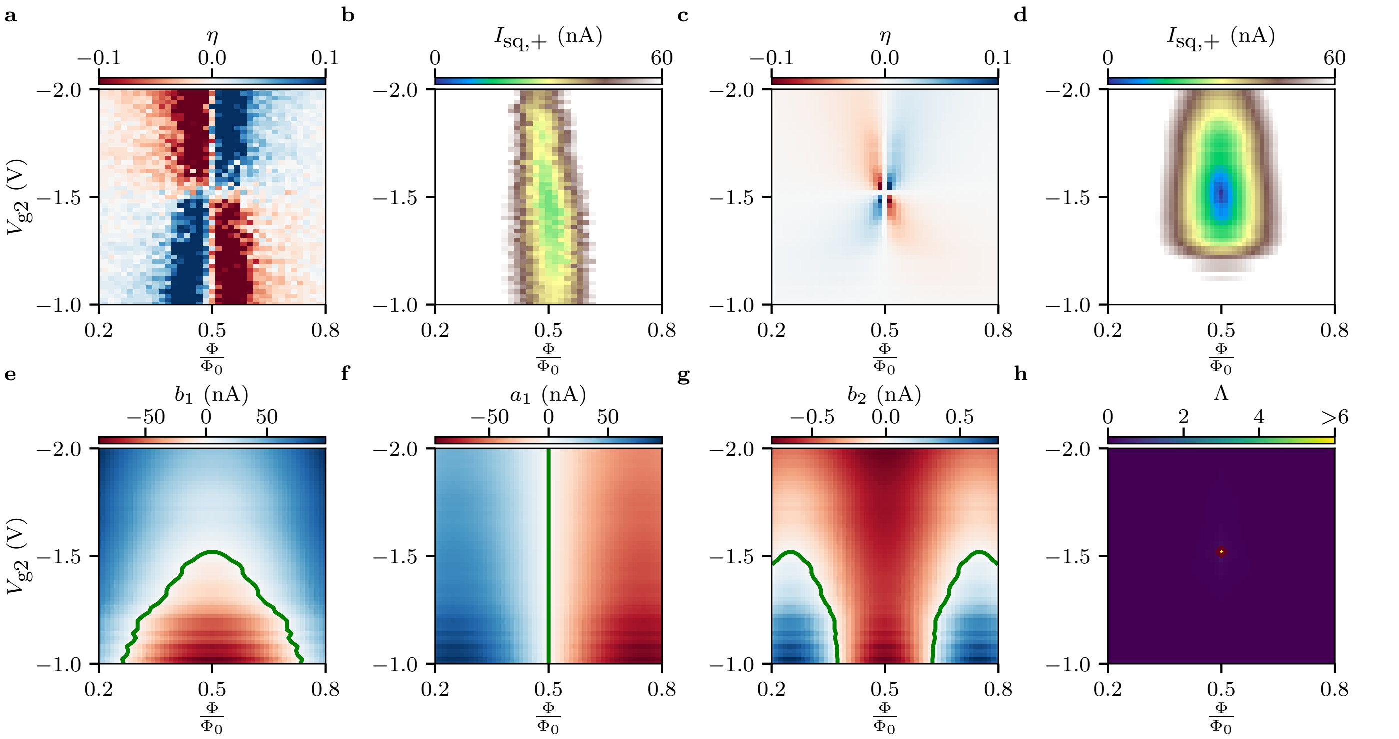

Figure S5: **Expected results with standard sinusoidal CPRs and low inductance ($L = 110\,\mathrm{pH}$)** **a** [**b**] shows the same plot as Fig. 4**e** [Fig. 4**f**]. **c** [**d**] Theoretical calculation of **a** [**b**], assuming standard sinusoidal CPR and 110 pH. The theoretical prediction does not resemble the experimental data. In **c**, $|\eta|$ is much smaller than what has been experimentally observed (**a**) and η is finite only slightly away from sweet spot, located at $V_{\mathrm{g2}} = -1.5\,\mathrm{V}$ and at $\Phi = 0.5\Phi_0$. In addition, the expected $I_{\mathrm{sq},+}$ is much lower than the measured values, especially at the sweet spot. In the theoretical calculation, $I_{\mathrm{sq},+} \approx 0$ at the sweet spot (**d**); whereas in the experimental data $I_{\mathrm{sq},+} \approx 20\,\mathrm{nA}$ (**b**). **e-f** First-order harmonics contribution as a function of I_{ret2} and Φ extracted using the same parameters as in **c-d**. Like for the case described in the main text, at the balanced point and at $\frac{\Phi}{\Phi_0} = 0.5$ both first harmonic terms vanish. **g** Second sinusoidal harmonics contribution as a function of I_{ret2} and Φ. Importantly, b_2 never vanishes at $\frac{\Phi}{\Phi_0} = 0.5$. Despite the fact that it is qualitatively similar to the situation of the main text (Fig. 4**k**), there is a major difference. At the sweet spot $|b_2| \approx 0.1$ nA, while in Fig. 4**k** $|b_2| \approx 20$ nA. **h** Ratio between second and first harmonic. For this situation, the second component would dominate the CPR just at the sweet spot.

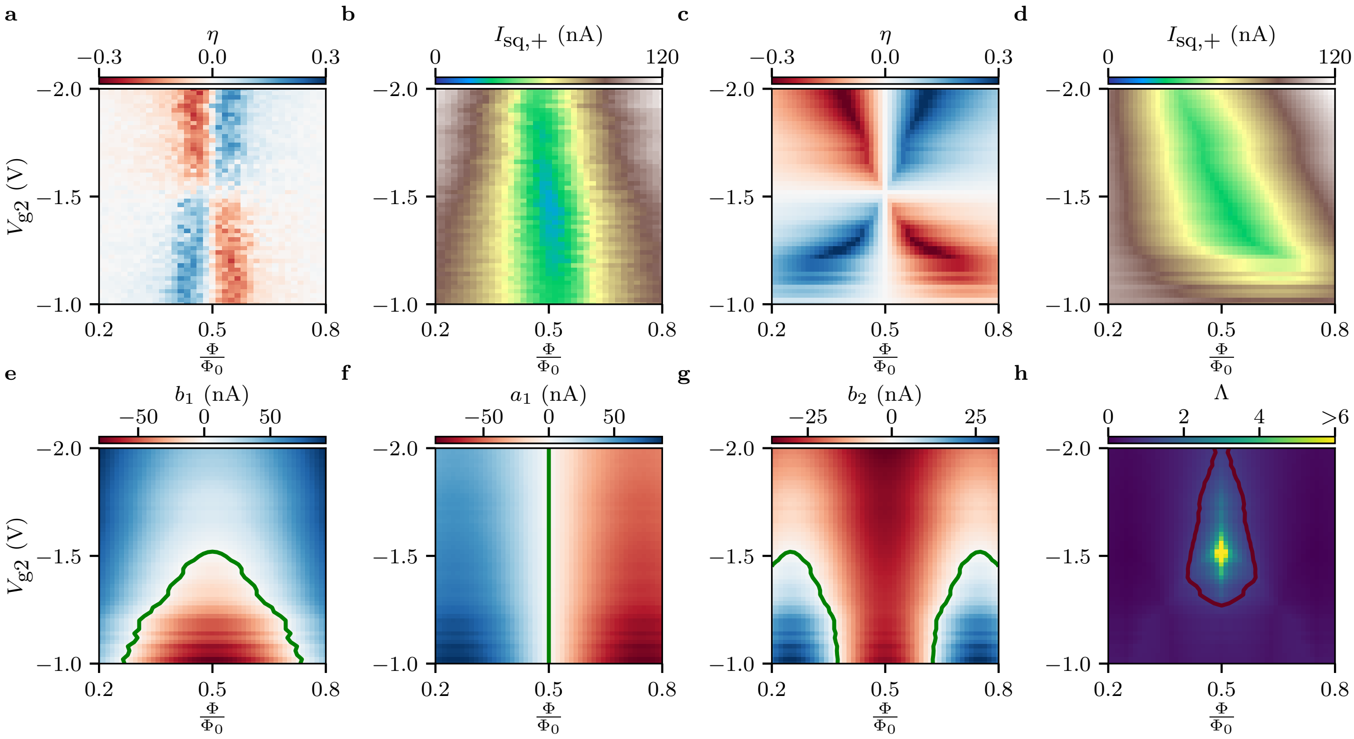

Figure S6: **Expected results with standard sinusoidal CPRs and with an inductance ($L = 6\,\text{nH}$) more than one order of magnitude higher than what was measured.** **a** [**b**] shows the same plot as Fig. 4**e** [Fig. 4**f**]. **c** [**d**] Theoretical calculation of **a** [**b**], assuming standard sinusoidal CPRs and $L = 6\,\text{nH}$, which is much higher than what has been experimentally estimated. The theoretical calculation resembles qualitatively the experimental data. **e-f** First-order harmonics contribution as a function of V_{g2} and Φ extracted using the same parameters as in **c-d**. Similar to the case described in the main text, close to the balanced point and at $\frac{\Phi}{\Phi_0} = 0.5$ both first harmonic terms vanish. **g** Second sinusoidal harmonics contribution as a function of V_{g2} and Φ. Importantly, b_2 never vanishes at half flux quantum. **h** Ratio between second and first harmonic. For this situation, the second component would dominate the CPR not only at the sweet spot, but also in the area enclosed by the red line corresponding to $K = 1$.

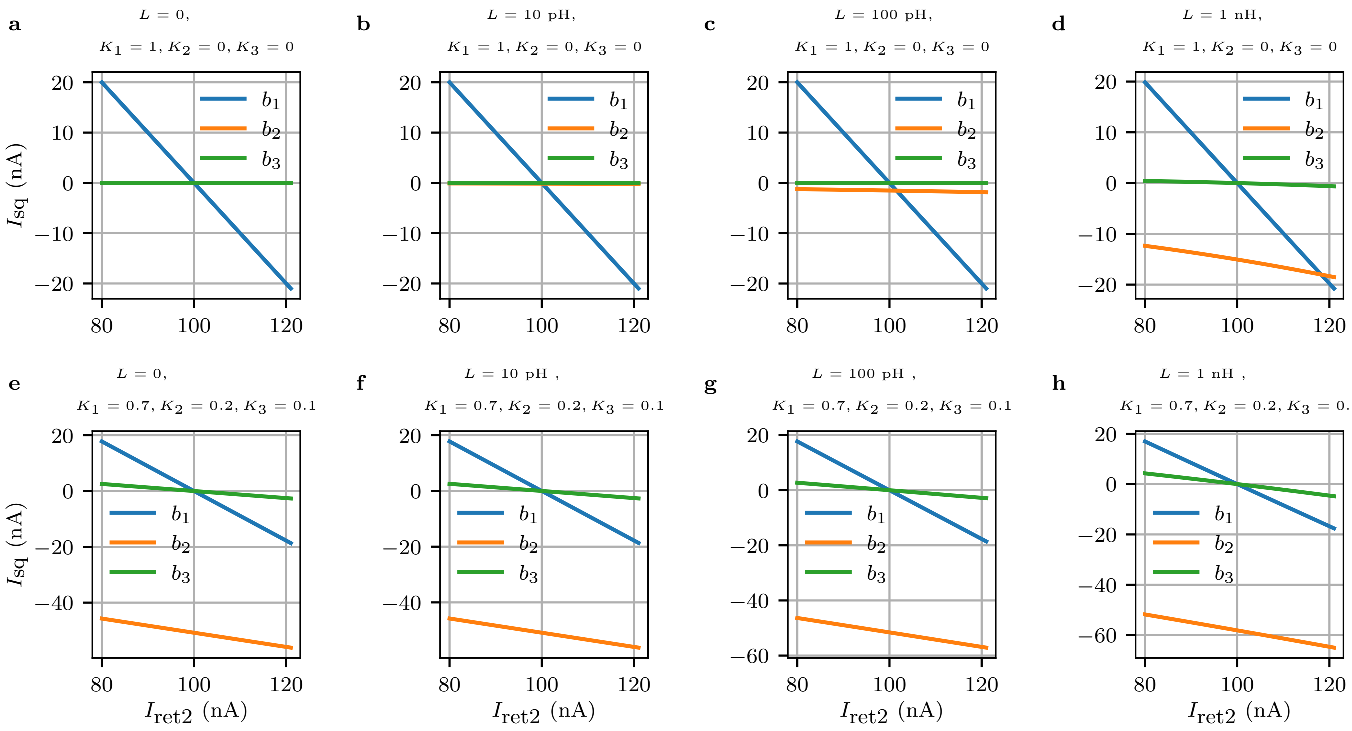

Figure S7: **Calculation of the first three harmonics of the SQUID CPR at half flux quantum by varying $I_{\mathbf{ret2}}$ and with $I_{\mathbf{ret1}} = 100$ nA.** In panels **a-d**, higher order term are neglected ($K_2 = K_3 = 0$). In panels **e-h**, higher order terms are considered ($K_1 = 0.7, K_2 = 0.2, K_3 = 0.1$). In the first column it is assumed $L = 0$, in the second $L = 10$ pH (as for the thick-Aluminum samples), in the third $L = 100$ pH (as for the thin-Aluminum samples) and in the fourth row $L = 1$ nH.

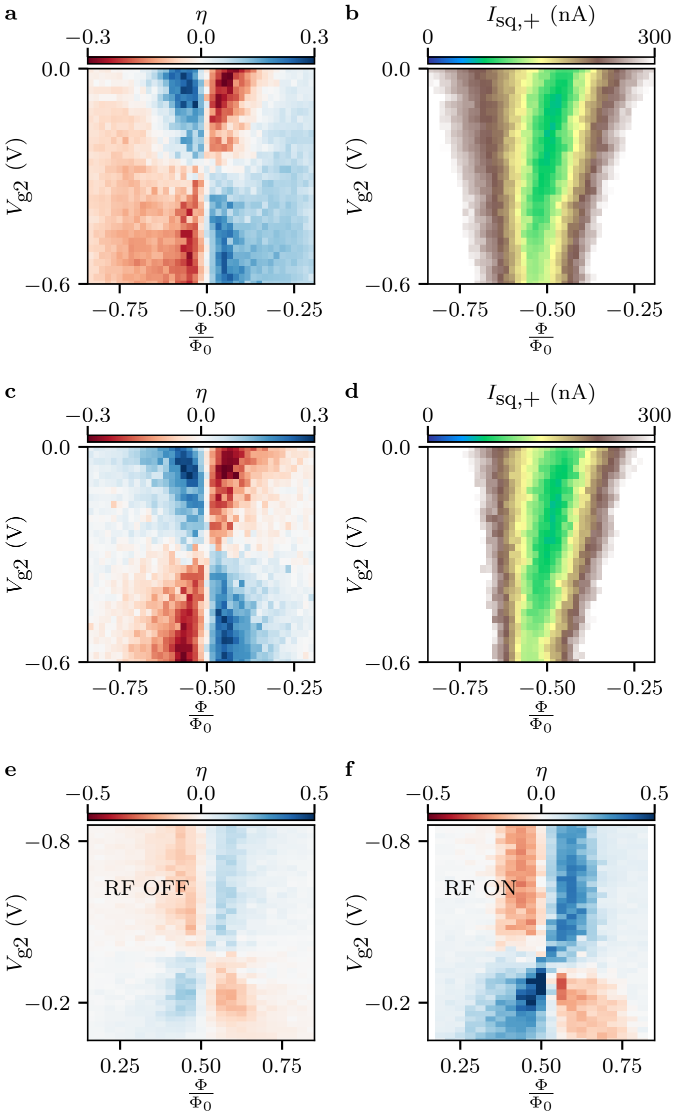

Figure S8: **Additional SDE results.** **a** [**b**] η [$I_{\text{sq},+}$] as a function of Φ and V_{g2} for a 30 nm-thick Al device. In **a** η is always zero at $\frac{\Phi}{\Phi_0} = 0.5$, independently on V_{g2}, and the polarity of the diode is inverted at the balanced point. Differently from Fig. 4**e**, the SDE does not vanish for all values of Φ at the balanced point. This can be due to an asymmetric harmonic contribution (see Fig. S3**c**). In **a** [**b**], η [$I_{\text{sq},+}$] was extracted by recording the retrapping current for both branches. **c** [**d**] is the same measurement as **a** [**b**] but now the switching current was recorded. Importantly, both approaches give similar results. **e** η as a function of Φ and V_{g2} for another sample D8 with thin film of Al deposited at low temperature. **f** same as **e** but with the addition of an applied RF power, namely with $P = -40\,\text{dBm}$ and $f_{\text{ac}} = 1.5\,\text{GHz}$. Importantly η increases with an applied drive.

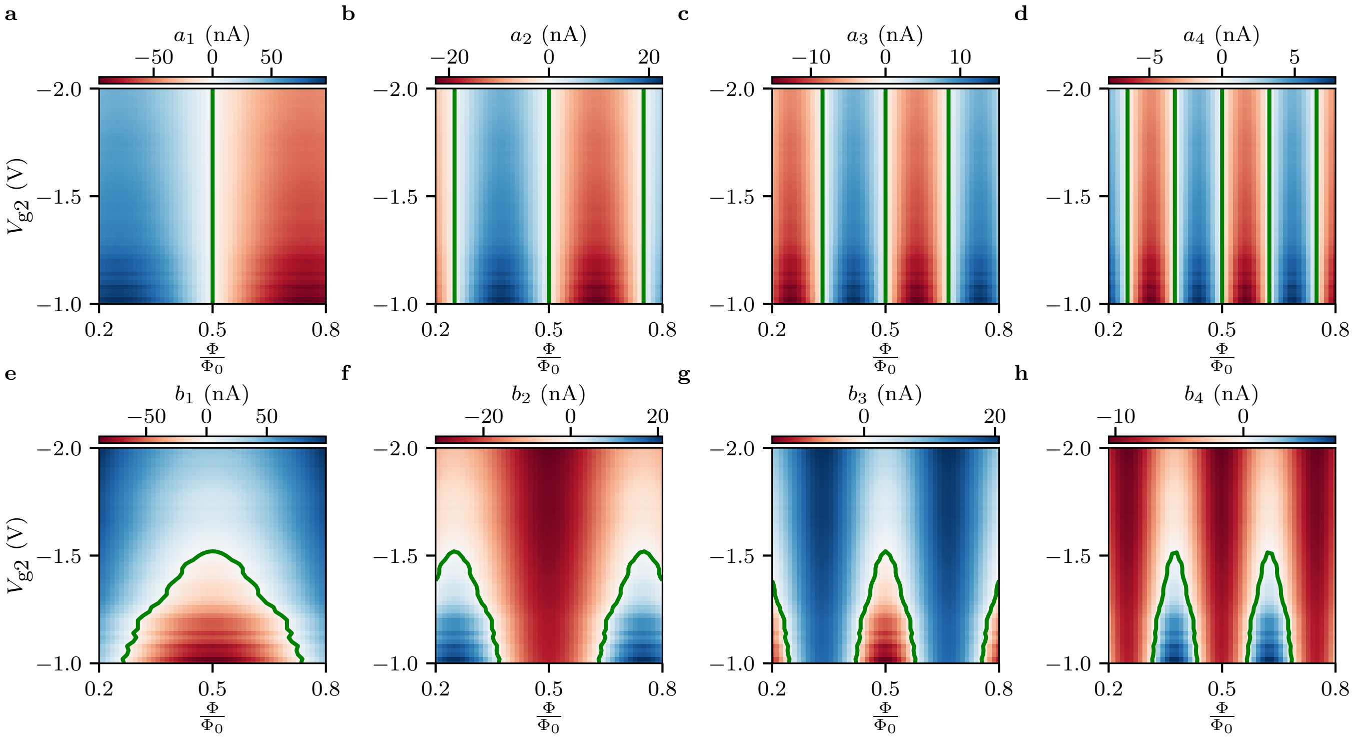

Figure S9: **Higher harmonic contribution as a function of Φ and $V_{\mathbf{g2}}$ estimated using the same parameters as in Fig. 4.** **a-d** [**e-h**] panels show the cosinus [sinus] terms. Interestingly, at the sweet spot ($V_{g1} = V_{g2}$ and $\frac{\Phi}{\Phi_0} = 0.5$) all terms, but b_2 and b_4, vanish.

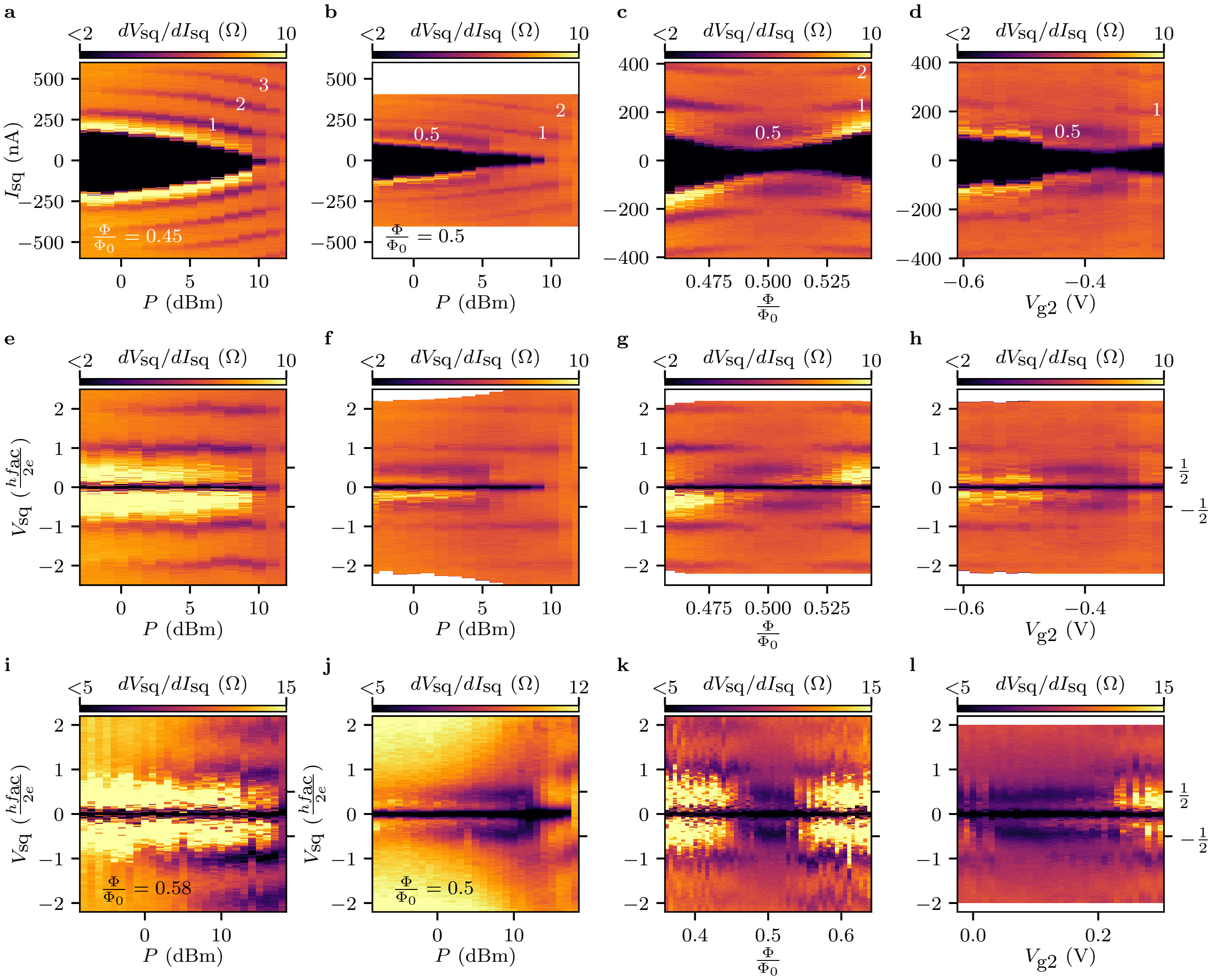

Figure S10: **Observation of half-integer steps for another device and identification of Shapiro steps.** **a** [**b**] Shapiro pattern for a 30 nm thick room temperature deposited Al sample with $R_\mathrm{shunt} = 10\,\Omega$ in the balanced regime ($I_\mathrm{ret1} \approx I_\mathrm{ret2} \approx 400\,\mathrm{nA}$) with $f_\mathrm{ac} = 500\,\mathrm{MHz}$ and at $\frac{\Phi}{\Phi_0} = 0.45$ [$\frac{\Phi}{\Phi_0} = 0.5$]. The differential resistance $dV_\mathrm{sq}/dI_\mathrm{sq}$ is plotted as a function of the RF power P and I_sq. Dips in $dV_\mathrm{sq}/dI_\mathrm{sq}$ correspond to integer Shapiro steps. Importantly at half flux quantum (**b**), the first half-integer step appears for low P, see white numbers. **c** Shapiro map as a function of I_sq and Φ in the balanced regime (like for the previous plots) for $P = 4\,\mathrm{dBm}$. The half-integer steps appear only close to $\frac{\Phi}{\Phi_0} = 0.5$. **d** Shapiro map as a function of I_sq and V_g2 for $P = 4\,\mathrm{dBm}$ and at $\frac{\Phi}{\Phi_0} = 0.5$. The half-integer step appears when the SQUID is close to the balanced condition, i.e. if $I_\mathrm{ret2} \approx 400 \pm 25\mathrm{nA}$. The white numbers in **c** and in **d** emphasize the appearance of the half-integer step. The second row corresponds to the same measurements as the first row but they are plotted as a function of V_sq instead of I_sq. The third row corresponds to the device shown in the main text in Fig. 5 but the data are plotted as a function of V_sq as well. **i** corresponds to Fig. 5**b**, **j** corresponds to Fig. 5**d**, **k** corresponds to Fig. 5**e** and **l** corresponds to Fig. 5**f**. V_sq is obtained by integrating the signal of $dV_\mathrm{sq}/dI_\mathrm{sq}$.

Supplementary References

[1] Probst, S., Song, F., Bushev, P. A., Ustinov, A. V. & Weides, M. Efficient and robust analysis of complex scattering data under noise in microwave resonators. *Review of Scientific Instruments* **86**, 024706 (2015).
